# Gene expression response in target organ and whole blood varies as a function of target organ injury phenotype

**DOI:** 10.1186/gb-2008-9-6-r100

**Published:** 2008-06-20

**Authors:** Edward K Lobenhofer, J Todd Auman, Pamela E Blackshear, Gary A Boorman, Pierre R Bushel, Michael L Cunningham, Jennifer M Fostel, Kevin Gerrish, Alexandra N Heinloth, Richard D Irwin, David E Malarkey, B Alex Merrick, Stella O Sieber, Charles J Tucker, Sandra M Ward, Ralph E Wilson, Patrick Hurban, Raymond W Tennant, Richard S Paules

**Affiliations:** 1 Cogenics, a Division of Clinical Data, Inc., Morrisville, NC 27560, USA; 2 NIEHS Microarray Group, National Institute of Environmental Health Sciences, National Institutes of Health, Research Triangle Park, NC 27709, USA; 3 Integrated Laboratory Systems, Inc., Research Triangle Park, NC 27709, USA; 4 National Toxicology Program, National Institute of Environmental Health Sciences, National Institutes of Health, Research Triangle Park, NC 27709, USA; 5 Biostatistics Branch, National Institute of Environmental Health Sciences, National Institutes of Health, Research Triangle Park, NC 27709, USA; 6 National Center for Toxicogenomics, National Institute of Environmental Health Sciences, National Institutes of Health, Research Triangle Park, NC 27709, USA; 7 Laboratory of Respiratory Biology, National Institute of Environmental Health Sciences, National Institutes of Health, Research Triangle Park, NC 27709, USA; 8 Laboratory of Experimental Pathology, National Institute of Environmental Health Sciences, National Institutes of Health, Research Triangle Park, NC 27709, USA; 9 Cancer Biology Group, National Institute of Environmental Health Sciences, National Institutes of Health, Research Triangle Park, NC 27709, USA; 10 Environmental Stress and Cancer Group, National Institute of Environmental Health Sciences, National Institutes of Health, Research Triangle Park, NC 27709; 11 Current address: Institute for Pharmacogenomics and Individualized Therapy, University of North Carolina at Chapel Hill, Chapel Hill, NC 27599, USA

## Abstract

This report details the standardized experimental design and the different data streams that were collected (histopathology, clinical chemistry, hematology and gene expression from the target tissue (liver) and a bio-available tissue (blood)) after treatment with eight known hepatotoxicants (at multiple time points and doses with multiple biological replicates). The results of the study demonstrate the classification of histopathological differences, likely reflecting differences in mechanisms of cell-specific toxicity, using either liver tissue or blood transcriptomic data.

## Abstract 2

Histopathology, clinical chemistry, hematology and gene expression data were collected from the rat liver and blood after treatment with eight known hepatotoxins.

## Background

The use of genomic approaches to better understand the adverse effects of environmental and xenobiotic exposures on human injury and disease processes engendered a great deal of early enthusiasm and excitement. This research initially focused on using gene expression alterations as measured by microarray analyses and is often referred to as 'toxicogenomics' [1]. Quite early on, investigators were able to demonstrate that exposure to different toxicants could be discriminated or classified in rodent model systems by microarray profiling of gene expression alterations in the target tissues, that is, tissues that display visible adverse effects in response to toxicant exposure [2-5].

Gene expression microarrays have developed over the past decade into a powerful tool for investigating biological, mechanistic, and disease processes in addition to developing genomic classifiers. Recent standardization efforts by the Microarray Quality Control Consortium, the Toxicogenomics Resource Consortium as well as other groups have clearly demonstrated the reproducibility of transcript level data generated using these approaches [6-9]. However, in most instances these studies have understandably been based on reference samples with little or no biological significance. The Microarray Quality Control Consortium did substantiate their findings by performing a cross-platform study using samples from a multi-agent rat toxicogenomics study at a single dose and time point and the Toxicogenomics Resource Consortium did perform a cross-laboratory, time course assessment using samples from a single toxic agent [10,11]. However, there are still open questions regarding the utility and applicability of the microarray technology in biological research and in particular with respect to understanding and classifying injury processes that arise as a consequence of exposures to various agents. For example, can gene expression data distinguish similar biological responses that occur in different physiological regions within an organ (for example, necrosis within different zones of the liver lobule) or similar lesions that are the result of exposure to different compounds?

Linking gene expression changes with more traditional toxicological measurements of adverse biological responses to toxicants (for example, histopathology and clinical chemistry), referred to as 'phenotypic anchoring', allowed investigators to gain new insight into the processes involved in the adverse effects on target tissues [12-15]. In addition to analysis of target tissues, the use of whole blood as a tissue source for gene expression profiling is extremely appealing and already has been demonstrated for a variety of diseases and exposures [16-22]. This has tremendous potential in a therapeutic setting - the use of blood as a surrogate for the primary tissue of interest greatly facilitates sample collection and analysis. The benefits would be realized in basic research studies as well. If transcript data in whole blood can function as a surrogate for the target organ, a researcher would be able to collect serial time points from an animal as opposed to harvesting tissue at a single time point after sacrifice. This would not only decrease the number of animals being used in a study, but would increase the amount and value of the data generated from a single animal as early transcriptional events could be phenotypically anchored to histopathological observations or clinical chemistry data that were not observed until later time points within the same animal. The amount of total RNA required to perform microarray-based gene expression profiling from whole blood continues to decrease, thereby increasing the potential for practical applications. Thus, one question of interest is whether expression data from whole blood can serve as a surrogate for a target organ through either an ability to detect the same transcript changes or an ability to identify different transcript biomarkers with similar or enhanced classification utility.

While much progress has been made in the application of toxicogenomics to the classification of toxicants and the investigation of mechanisms of toxicity, a full realization of its potential in a systems biology context, sometimes referred to as 'systems toxicology' [23], has yet to be accomplished. A primary obstacle has been the lack of truly robust data sets that capture not only genome-wide gene expression measurements but also traditional biological and toxicological information associated with exposures that vary over dose and time. This need was recently highlighted in the National Research Council's report on toxicogenomics [24]. Here we present a comprehensive, public dataset of gene expression and accompanying data (histopathology, clinical chemistry, hematology) from a standardized study to serve as a resource to further advance the development of systems toxicology. The present report details the experimental design and the different data that were collected, and provides examples of how these data can be used to address important biological questions. This investigation of eight known hepatotoxicants was designed to evoke acute liver injury with similarities as well as differences in the type and location of injury that result.

The eight compounds are all acute hepatotoxins that cause hepatocellular necrosis following a single administration at a suitable dose. Most of the compounds target hepatocytes; however, monocrotaline targets endothelial cells leading to hemorrhage and activation of the coagulation system. The toxicity of bromobenzene, 1,2- and 1,4-dichlorobenzene, N-nitrosomorpholine, monocrotaline, and thioacetamide require metabolic activation by various cytochrome P450s to reactive intermediates [25-29]. Since cytochrome P450 expression occurs primarily in the perivenous to centrilobular region of the hepatic plate, these agents generally damage the centrilobular region of the liver. The toxicity of diquat is associated with a one electron reduction/oxidation reaction that is catalyzed by NADPH cytochrome c reductase and leads to the production of high levels of reactive oxygen species [30]. The mechanism responsible for the toxicity of galactosamine is not as well understood but is thought to be associated with depletion of UTP caused by the conversion of galactosamine to UDP-hexosamines and UDP-N-acetylhexosamines [31].

The work was performed in its entirety using standardized procedures for the in-life work and for the generation of gene expression microarray data (n = 1,704 hybridizations). Doses that ranged from 'sub-toxic' to 'toxic' exposures were selected. Additionally, gene expression profiling was performed on two commercially available platforms (Agilent and Affymetrix) for one of the tissues (liver), thereby providing an opportunity to corroborate findings across platforms. In-life observations were recorded and clinical chemistry, hematology, and liver histopathology were also assessed for all 426 animals. These additional data facilitate the phenotypic anchoring of the gene expression data and confirm that the goal of the study to evoke different types of liver injury was achieved. Gene expression levels in whole blood were also assayed to evaluate whether biomarkers of different types of liver damage could be identified in this readily accessible bio-fluid.

The analyses presented here further demonstrate the utility of using microarrays as a tool for gene expression profiling to address biological questions. Specifically, this experiment was designed to provide a means to generate mechanistic and predictive measures of toxicity by integrating multiple data streams that were recorded within this standardized study. These model chemical exposure studies also provide a comprehensive data set with a well defined phenotypic anchor (liver injury) that is needed to assess the utility of gene expression profiling from whole blood samples. These data can now serve as a rich resource to the scientific community to test and validate these as well as other hypotheses, and have been made freely available in a public repository [32-34]. While the experimental design is based on a common model for toxicogenomics studies, the findings and/or the results of mining these data can be applied to other fields using microarrays as a research tool.

## Results and discussion

### Experimental design

The motivation for this study was the need for a rich, public dataset consisting of multiple types of data (gene expression, histopathology, and so on) that could be interrogated to test hypotheses regarding the application of gene expression profiling data generated from microarrays. We therefore designed a study with multiple compounds (n = 8) that would induce a common phenotype (liver injury), while also differing in the extent and/or severity of the injury and/or the co-occurrence of other noted histopathological observations, such as the type of inflammatory cell infiltrates, the presence of bile duct hyperplasia, and so on (Table 1). The selected compounds are acute hepatotoxicants, requiring only a single administration at a suitable dose to elicit a toxic response. All eight compounds are rapidly absorbed from the gastrointestinal tract following administration, but are not direct acting in that they require biotransformation to toxic-reactive intermediates within the liver. From a preliminary dose finding study, three doses were selected corresponding to a 'sub-toxic', 'moderately toxic' and 'toxic' dose for each compound (Table 2). Separate groups of four to six animals were treated with compound at each of these doses or with a vehicle control at time 0, and then sacrificed either 6, 24, or 48 hours later. Blood and liver were harvested at the time of sacrifice for clinical chemistry, hematology, histopathology and gene expression profiling using standardized procedures across all compounds. Additional data file 1 details all of the measurements that were collected, except the gene expression profiling data, for all of the animals in this study. All data, including the gene expression data, are available through a publicly accessible website [34].

**Table 1:** Hepatotoxicants studied

Compound	Low dose 'sub-toxic' (mg/kg)	Medium dose 'moderately toxic' (mg/kg)	Medium high dose (mg/kg)	High dose 'toxic' (mg/kg)	Vehicle	Delivery method	Primary site of livery injury
Bromobenzene	25	75	NA	250	Corn oil	Oral gavage	Centrilobular
1,2-Dichlorobenzene	15	150	NA	1,500	Corn oil	Oral gavage	Centrilobular
1,4-Dichlorobenzene	15	150	NA	1,500	Corn oil	Oral gavage	NA*
N-nitrosomorpholine	10	50	NA	300	PBS	Oral gavage	Centrilobular
Diquat	5	10	20	25	PBS	Intraperitoneal injection	Mid-zonal to centrilobular
Monocrotaline	10	50	NA	300	PBS	Oral gavage	Centrilobular
Thioacetamide	15	50	NA	150	PBS	Oral gavage	Centrilobular
Galactosamine	25	100	NA	400	PBS	Intra-peritoneal injection	Random

*1, 4-Dichlorobenzene was intended to function as a non-toxic analog of 1,2-dichlorobenezene; however, centrilobular hepatocyte necrosis was observed in one of the six low dose animals at 6 hours and all four of the high dose animals at the 24 hour time point. NA, not applicable; PBS, phosphate-buffered saline.

**Table 2:** Summary of histopathological diagnoses and severities

	Severity				
	
Histopathological diagnosis	None	Minimal	Mild	Moderate	Marked
Centrilobular hepatocyte necrosis	304	65	15	8	26
Centrilobular, mid-zonal hepatocyte necrosis	399	8	5	4	2
Mid-zonal hepatocyte necrosis	417	1	0	0	0
Focal hepatocyte necrosis	392	11	12	2	1
Centrilobular cellular infiltrates	333	50	17	13	5
Centrilobular, mid-zonal cellular infiltrates	400	5	8	5	0
Portal cellular infiltrates	394	16	5	3	0
Focal cellular infiltrates	370	34	10	4	0
Depletion glycogen	324	19	33	16	26
Centrilobular hepatocyte degeneration	403	9	3	3	0
Centrilobular, mid-zonal hepatocyte degeneration	402	4	7	5	0
Hepatocyte apoptosis	410	7	1	0	0
Hepatocyte hypertrophy	382	10	16	0	10
Hepatocyte fatty change	416	2	0	0	0
Bile duct hyperplasia	405	11	2	0	0
Congestion	397	10	5	6	0
Hemorrhage	384	34	0	0	0
Hepatocyte regeneration	412	3	0	2	1
Mitosis	393	12	5	4	4

The expected result of exposure to both the moderately toxic and toxic doses of these compounds (except for 1,4-dichlorobenzene) was necrosis within the liver lobule. For most compounds it was expected that the necrosis would be localized to the centrilobular region, whereas with diquat the damage was expected to extend into the midzonal region, and with galactosamine it was expected to be randomly distributed throughout the liver. The histopathology data confirmed these expectations; however, some individual animal variability was observed (Table 1 and Additional data file 1). Variability in the evoked phenotypic response is commonly observed in these types of in-life studies, despite conducting the experiments in an in-bred population using highly standardized procedures. Although such variability can be confounding when the data are analyzed using dose, time, and compound to organize groups, here we consider this variability to be an asset in that it provided an opportunity to examine molecular responses and mechanisms not only according to the mode of action of the included compounds, but also according to the severity and character of the response irrespective of inducing compound. Furthermore, this variability in response provides an opportunity to identify biomarkers that correlate with hyper- and hyposensitivity in individual animals.

### Compound-specific responses

Previous work has demonstrated that toxicants can be classified using gene expression data derived from the primary target tissue [2,3,35,36]. The compounds in this study were chosen based on their ability to induce acute hepatotoxicity and the similarities and differences in the type and location of the damage resulting from exposure. Since the compounds in this study are acute hepatotoxicants resulting in hepatocyte necrosis, we wanted to corroborate these findings with chemicals that could be considered closely related based on the damage they evoke.

For each compound there are three doses and three time points for a total of nine dose/time groups with four to six animals in each group (the additional dose for diquat was omitted from these analyses). A support vector machine (SVM) approach was used to identify compound-specific classifiers within each dose/time group for each tissue type (blood and liver). The resulting classifiers (Additional data file 2) were then used to group samples based on principal component analyses and hierarchical clustering to determine whether or not the classifiers functioned well at separating all of the animals within a dose/time group into different compound groups.

**Figure 1:**
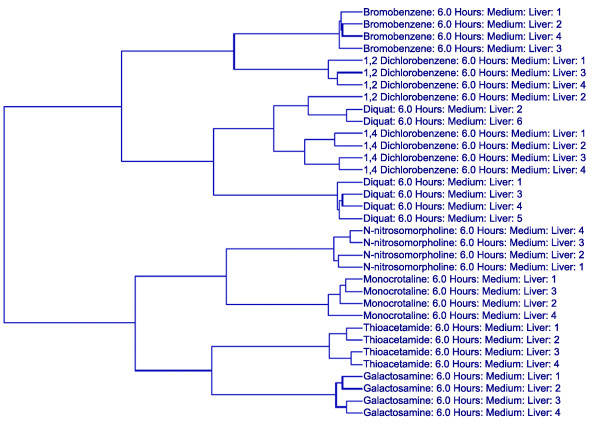
Hierarchical clustering of animals in the medium dose/6 hour group using SVM-derived classifiers. Two-way hierarchical clustering using Ward's minimum variance as the heuristic criteria and Euclidean distance as the similarity metric was performed on all of the animals in the medium dose/6 hour group using the liver expression values for the 160 transcripts identified as compound classifiers for this dose/time group by a SVM algorithm. The degree of relatedness between each sample is represented by the dendrogram (hierarchical tree) presented in this figure, wherein the height of each branch represents the distance between the two objects being connected.

An SVM identified 160 transcripts for the classification of the 8 compounds within the liver profile data of animals treated with the medium ('toxic') dose and sacrificed at the 6 hour time point. When the expression level data for these transcripts were used to cluster the medium dose samples at the 6 hour time point, the classifiers separated the animals into the eight different compound groups, with the exception of three individual animals (two diquat animals (animals 2 and 6) and one 1,2-dichlorobenzene animal (animal 2); Figure 1). Interestingly, these animals had a noticeably different phenotypic response compared to the other individuals in their compound group or represent a compound group demonstrating significant diversity in the manifested phenotype. For example, three of the animals in the 1,2-dichlorobenzene group (animals 1, 3, and 4) had minimal levels of hepatocellular necrosis at this dose and time; however, the sample that did not cluster (animal 2) with these animals did not have any observable necrosis. The diquat treatment at this dose and time point resulted in variability in regards to the phenotypic response at the level of histopathology. Three of the six diquat treated animals had no apparent histopathological observations, two had noted levels of glycogen depletion and one animal did have minimal levels of hepatocellular necrosis. While the grouping of the samples in the cluster does not perfectly reflect the three different levels of liver damage noted in this compound group, the lack of grouping is consistent with the variability in regards to the treatment response. This confirms that while transcript data from the primary target organ can effectively classify compounds, this is true only inasmuch as the treatment evokes a level of consistency in the response. In instances in which the response to a particular dose of a given compound is variable in regards to the phenotype that is evoked, the transcript expression levels are more reflective of the phenotype as opposed to the inducing compound. These findings underscore the importance of phenotypical anchoring.

When one considers the variability of the presence and severity of necrosis and the infiltration of inflammatory cells, it is not surprising that gene expression data from a target organ would not accurately group all of the samples within a given compound. For this reason, the gene expression data generated from whole blood were examined to determine if these data could more accurately group samples based on compound. Blood is a highly accessible bio-fluid, yet is not the primary target tissue for compound toxicity. When the liver data were examined, the SVM-identified classifiers for each of the nine dose/time groups were not able to accurately group all of the individuals into the eight compound groups. In contrast, the classifiers that were generated using the blood data were able to accurately group all of the individuals according to the eight compounds in only one of the nine dose/time groups, but performed better than the liver data at grouping individuals into compound groups in six of the nine dose/time groups. Table 3 indicates the number of classifiers that were identified for each dose/time group for each tissue type (as well as the number of classifiers overlapping across the tissue types within a dose/time group) and indicates the accuracy each set of classifiers achieved in grouping all of the animals into compound groups. Interestingly, the SVM classifiers generated from blood data outperformed the SVM classifiers generated from liver data in six of the nine dose/time groups; the exceptions were the low dose, 6 hour group and the low and high dose groups for the 48 hour time point. Though the number of common classifiers across the tissue types within a given dose/time group were small, this was not an unexpected finding considering that the target tissue is the site of the injury whereas the blood represents a readily available tissue source functioning as a surrogate (Table 3). Cumulatively, these findings reinforce the utility of performing gene expression profiling experiments on blood as a suitable surrogate for the target organ.

**Table 3:** SVM classifier accuracy

		Blood				Liver			
			
Time (Hours)	Dose	Number of animals in the dose/time groups	Animals correctly grouped by compound	Percentage grouped correctly	Number of classifiers	Animals correctly grouped by compound	Percentage grouped correctly	Number of classifiers	Overlap of classifiers
6	Low	34	27	79.4	160	32	94.1	110	12
6	Medium	34	32	94.1	210	31	91.2	160	17
6	High	34	30	88.2	10	23	67.6	10	0
24	Low	34	33	97.1	110	28	82.4	310	16
24	Medium	34	34	100.0	360	30	88.2	60	12
24	High	34	32	94.1	360	30	88.2	10	0
48	Low	34	30	88.2	210	32	94.1	360	22
48	Medium	34	33	97.1	310	32	94.1	460	36
48	High	28	22	78.6	60	27	96.4	10	1
			Average	90.8		Average	88.5		

**Figure 2:**
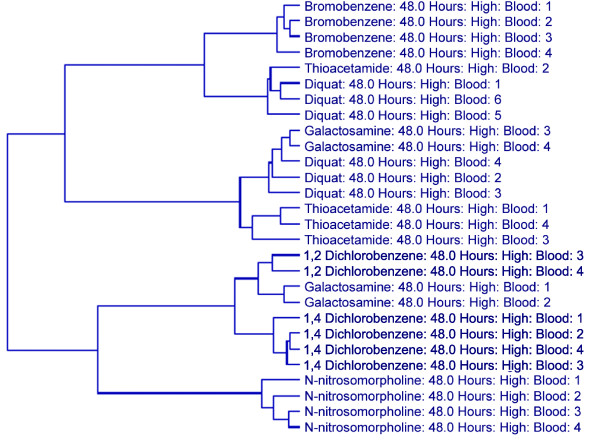
Hierarchical clustering of animals in the high dose/48 hour group using SVM-derived classifiers. Two-way hierarchical clustering using Ward's minimum variance as the heuristic criteria and Euclidean distance as the similarity metric was performed on all of the animals in the high dose/48 hour group using the blood expression values for the 60 transcripts identified as compound classifiers for this dose/time group by a SVM algorithm. The degree of relatedness between each sample is represented by the dendrogram (hierarchical tree) presented in this figure, wherein the height of each branch represents the distance between the two objects being connected.

Transcript data from blood, however, were also unable to group individual animals in some instances in which the compound manifested different phenotypic responses across the animals of that particular dose/time group. At the high dose/48 hour time point, three of the compounds (diquat, thioacetamide, and galactosamine) demonstrated variability in regards to the extent of hepatocellular necrosis, the amount of inflammatory cell infiltrates and/or indications of hepatocellular repair (mitosis). Consistent with this observation, these are also the three compounds that did not separate perfectly in the dendrogram resulting from the hierarchical clustering of the blood gene expression data for the 60 SVM-identified classifiers for this dose/time group (Figure 2), suggesting that the phenotypic response resulting from exposure to a given compound was having a greater impact on the gene expression profile compared to a unified transcriptional response resulting merely from the exposure to the compound *per se*. Taken together, these results indicate that the details of the injury resulting from compound application are a more appropriate grouping than are the doses and time points of individual compounds when using gene expression data from the primary site of injury - in this case the liver - or when using gene expression data from a surrogate source such as whole blood.

**Figure 3:**
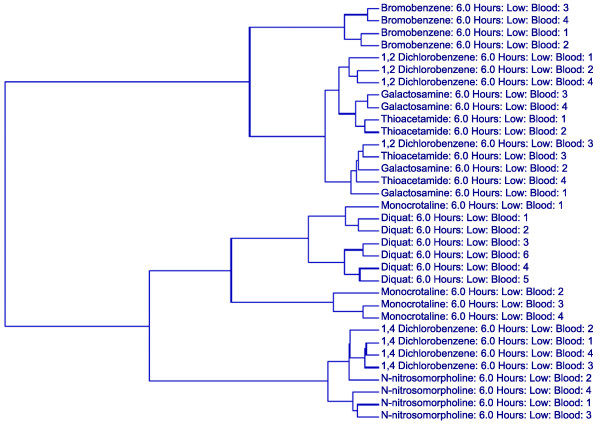
Hierarchical clustering of animals in the low dose/6 hour group using SVM-derived classifiers. Two-way hierarchical clustering using Ward's minimum variance as the heuristic criteria and Euclidean distance as the similarity metric was performed on all of the animals in the low dose/6 hour group using the blood expression values for the 160 transcripts identified as compound classifiers for this dose/time group by a SVM algorithm. The degree of relatedness between each sample is represented by the dendrogram (hierarchical tree) presented in this figure, wherein the height of each branch represents the distance between the two objects being connected.

Another illuminating trend in both liver and blood gene expression data was the grouping of galactosamine and thioacetamide samples. For example, using the 160 SVM-identified classifiers generated from the blood data for the low dose/6 hour samples, most of the samples within a given compound group clustered together (Figure 3). It was not surprising to observe several cases in which a specific animal did not group with the others from a compound group since virtually no histopathological phenotypes were observed at this time point after exposure to a low dose. In fact, in most instances this exposure dose did not elicit any injury at any of the time points. However, the galactosamine and thioacetamide samples grouped together. Interestingly, these are the only two compounds that evoked biliary hyperplasia in this study, albeit at a later time point (48 hours). Their grouping in the low dose/6 hour samples suggests that they activate similar transcriptional responses long before a common visible phenotype is observed, despite the fact that these compounds are not structurally related.

In order to assess the biological themes within each set of classifiers, a Gene Ontology (GO) enrichment analysis was performed on the classifiers for each tissue/time point/dose group using GoMiner [37,38]. The enrichment *p*-values for all GO categories that had at least five members measured by the microarray are detailed in Additional data file 3. The GO categories that were found to be enriched in multiple tissue/time point/dose groups revealed some interesting aspects of the biology underlying the molecular response to the exposure to these acute hepatotoxicants. Not surprisingly, categories like 'response to chemical stimulus' and 'response to wounding' were found to be enriched in many of these groups. 'Taxis' and 'chemotaxis' were the most prevalently enriched GO categories in both tissue types, which likely reflects the differential response with regards to the inflammatory cell infiltrates across the compounds. 'Wound healing' was significantly enriched in many of the time point/dose groups in the liver, except at the time points associated with the high dose exposure, which is consistent with the histopathological observations with the sub-toxic and moderately toxic doses. Interestingly, this category was enriched at the 24 and 48 hour time points for the high dose exposures in blood. Also surprising was the observation that classifiers annotated to 'wound healing' were different for each of these time points in blood, potentially suggesting that the differences in the identity of the genes may represent some of the temporal responses to the healing and repair processes that occurred between the 24 and 48 hour time points.

Cumulatively, these results support the previous findings that toxicants can be classified and differentiated based on gene expression profiling data from the target organ [2,3,35,36], but extends our understanding by determining that such results can also be obtained using whole blood. Importantly, in instances where a specific dose of a compound resulted in variable severity of liver injury, it was found that the specifics of the injury were a more significant determinant of expression levels than were compound-specific patterns of expression. This strongly reinforces the need to phenotypically anchor gene expression data in order to appropriately interpret the results.

### Transcript profiles of toxicity in whole blood

**Figure 4:**
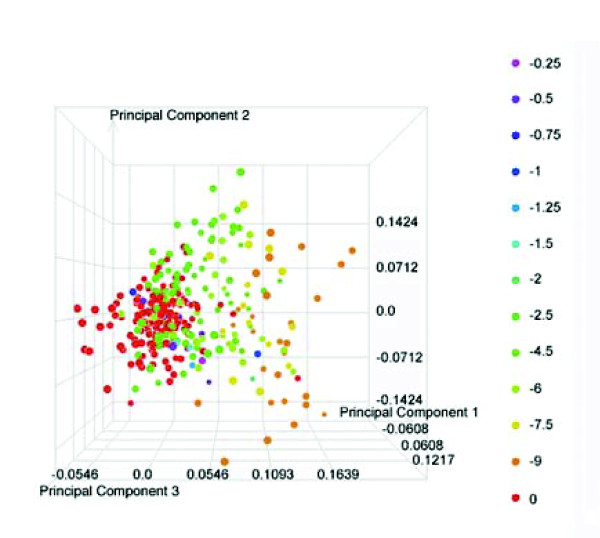
PCA of transcripts identified as differentially expressed across the 'Response to hepatocellular injury' category in blood. Blood expression data from the 30 transcripts identified as differentially expressed across the Response to hepatocellular injury category from all 318 treated rats were subjected to PCA. The first principal component is represented by the x-axis, while the second principal component is represented by the y-axis. Each individual animal is represented as a circle and the relationship between the color of the circle and the categorical scores for the Response to hepatocellular injury is illustrated in the key.

Having demonstrated the utility of performing gene expression profiling on whole blood samples as an effective surrogate for the target organ to classify compound exposures, we sought to further explore the utility of this readily available sample source by establishing whether blood transcript data could provide insights into the presence and severity of injury within a target organ upon toxicant exposure. In previously published work using these same in-life studies, we detailed an approach to compile histopathological diagnoses into common biological themes and to appropriately weigh and score these observations [39]. Using this approach, the diagnoses involving 'glycogen depletion', 'hypertrophy', 'fatty change', and 'necrosis' were combined in a 'Response to hepatocellular injury' category. For each individual animal in this study, a score was calculated for this category based on the absence or presence (and associated severity score) of the histopathological observation comprising this category (Additional data file 1). These scores were used as the factor level in a one-way analysis of variance (ANOVA) involving the gene expression data from whole blood. A total of 30 transcripts was found to vary significantly (*p*-value ≤ 1 × 10^-7^) across the factor levels. Interestingly, over 60% of these transcripts (n = 19) overlap with the set of 3,659 transcripts that were identified using the same ANOVA approach with the liver gene expression data [39]. The gene expression data from all 318 rats for this group of 30 transcripts were then used in a principal component analysis (PCA). The resulting visualization from this analysis (Figure 4) reveals that the variability contained in the first principal component (the combination of variables that explains the greatest amount of variation) provides good separation of the individuals based on their 'Response to hepatocellular injury' score as indicated by the increasing trend from left to right in the figure. The separation is not perfect, which is expected when one considers that this is only a portion of the biological response to treatment that these animals undergo. However, this clearly illustrates the power to be able to detect not only the presence of liver injury, but also an indication of severity using transcript data from whole blood.

## Conclusion

We have generated a data set that will be a unique and valuable resource for the environmental health and toxicological research community. The results of the study presented here demonstrate that, using this data set, differences in the phenotypic manifestation of diverse hepatotoxicants can be resolved by microarray analysis. It was possible to classify histopathological differences, most likely reflecting differences in mechanisms of cell-specific toxicity, using either liver tissue or blood. Such data may allow for mechanistic inference and lead to a more precise definition of the potential hepatotoxicity of new compounds. In addition, the availability of public access to the data derived from these studies provides a resource to other investigators pursuing these and related issues.

## Materials and methods

### Animals and animal care

Male Fischer 344/N rats approximately 10-12 weeks old were obtained from Taconic laboratory animals (Germantown, NY, USA). The animals were acclimated for 14 days and observed for absence of disease. All studies were conducted at Integrated Laboratory Systems, Inc. (Research Triangle Park, NC, USA). The rats were kept two per polycarbonate cage (Lab Products, Inc., Maywood, NJ, USA) with Sani-Chips bedding (PJ Murphy Forest Products Corp., Montville, NJ, USA). The animal rooms were maintained at 17-25°C and 33-96% relative humidity with a 12 hour dark-light cycle and 10 room air changes per hour. Cages were changed twice per week and NTP-2000 diet and tap water were provided *ad libitum*.

### In-life studies

Hepatotoxicants were identified based on published literature and chosen due to their ability to injure different cell types and regions of the liver. Doses were determined with pilot experiments. For each chemical, doses that elicited a sub-toxic ('low'), a moderately toxic ('medium') or a overtly toxic ('high') response 24 hours after treatment were selected. The doses, vehicle, method for delivery, and primary site of liver damage for each of the eight compounds are detailed in Table 1. For each compound, control animal groups were treated with vehicle alone. All animals were fasted for 12-18 hours prior to exposure. Groups of four to six animals were dosed between 8:30 and 9:30 a.m.; thereafter, feed was provided *ad libitum*. After 6, 24 or 48 hours of treatment, animals were anesthetized by isoflurane anesthesia. Whole blood was collected via retro-orbital plexus for hematology and gene expression analysis. Serum was collected for clinical chemistry. The animals were euthanized while under anesthesia by cervical dislocation and necropsied within 4 minutes of sacrifice. The left liver lobe was promptly removed and two defined cross sections were placed in 10% neutral buffered formalin for subsequent histopathology. The remainder of the left lobe was flash frozen for subsequent RNA isolation based on established methods [40]. Experiments were performed according to established guidelines and an approved Animal Study Protocol was on file prior to initiation of the study [41]. Additional data file 4 details the date of death, method of sacrifice, initial and final body weight along with gross findings/observations prior to and during necropsy for every animal. These data are also available in the Chemical Effects in Biological Systems (CEBS) knowledgebase under the CEBS ID# 001-00001-0020-000-4 [34].

### Histopathology

The cross sections that were fixed by immersion in 10% neutral buffered formalin (see 'In-life studies' section above) were transferred to histology grade alcohol for 18-24 hours after necropsy. Tissues were then processed, embedded in paraffin, sectioned at 5 microns, and stained with hematoxylin and eosin. A pathologist completed the initial microscopic evaluation of each liver sample for a given compound. Grading criteria for each diagnosis are detailed in Additional data file 5. An independent pathology quality assessment review was performed by a second pathologist on the initial histopathological findings. A third pathologist reviewed the quality assessment report and the slides and convened a consistent group of four to five pathologists (including the two mentioned above) to resolve any inconsistencies or discrepancies in diagnoses and grading for all studies. Additional data file 1 details the histopathological diagnoses and their associated severity scores for each animal in the study. These data are also available in CEBS under the CEBS ID# 001-00001-0020-000-4 [34].

### Clinical chemistry

At sacrifice, blood was collected in clotting tubes (BD Microtainer^® ^Tubes, BD, Franklin Lakes, NJ, USA) and serum was separated. Clinical chemistry analysis was performed on all rats using a COBAS MIRA (Roche Diagnostics, Montclair, NJ, USA) using commercially available reagents from Equal Diagnostics (Exton, PA, USA) for the following assays: total protein, albumin, cholesterol, creatinine, direct bilirubin, total bilirubin, total bile acid, blood urea nitrogen and triglycerides. In addition, an enzymatic activity for the following proteins was measured: alanine aminotransferase, alkaline phosphatase, aspartate aminotransferase, lactate dehydrogenase, and sorbitol dehydrogenase. Additional data file 1 details the clinical chemistry value for each of these parameters for every animal. These data are also available in CEBS under the CEBS ID# 001-00001-0020-000-4 [34].

### Hematology

Blood was collected in EDTA tubes (BD Microtainer^® ^Tubes). The samples were assayed using the Technicon H*1 hematology analyzer (Bayer Corporation, Tarrytown, NY, USA). The instrument performs white blood cell (WBC) counts by two independent methods. The values must have agreed within 10%. Manual counts and smear estimates were used to confirm values, when necessary. To monitor red blood cell (RBC) parameters, spun microhematocrits were performed for each specimen for comparison with the automated hematocrit (which is calculated from the directly measured RBCs and the mean cell volume). Control products were assayed after every tenth specimen. For WBC differential data, smears were Wright-Giemsa stained using an Ames Hema-Tek II automated slide stainer (Bayer Corporation, Ames Division, Elkhart, IN, USA) and manual WBC differential counts were performed on the first sample in each group. For reticulocyte counts, equal amounts of whole blood and New Methylene Blue stain were allowed to incubate at room temperature for at least 15 minutes. Smears were prepared and read to determine the percent reticulocytes per 1,000 RBCs. The percent value is multiplied by the RBC count to determine the absolute number of reticulocytes per microliter. Additional data file 1 details the values for each of these parameters for every animal. These data are also available in CEBS under the CEBS ID# 001-00001-0020-000-4 [34].

### RNA isolation and gene expression profiling

RNA was isolated from the flash frozen portion of the left lateral liver lobe using RNeasy Midi columns (Qiagen, Valencia, CA, USA) according to the manufacturer's protocol and also from whole blood using the PaxGene Blood RNA Kit (Qiagen). The quantity and purity of the extracted RNA was evaluated using a NanoDrop ND-1000 spectrophotometer (Nanodrop Technologies, Wilmington, DE, USA) and its integrity measured using an Agilent Bioanalyzer. For microarray hybridizations performed on the Agilent platform, 1 μg of total RNA from the left liver lobe or whole blood from either an individual rat or a pooled sample representing time-matched, vehicle control animals was amplified and labeled with a fluorescent dye (either Cy3 or Cy5) using the Low RNA Input Linear Amplification Labeling kit (Agilent Technologies, Palo Alto, CA, USA) following the manufacturer's protocol. The amount and quality of the fluorescently labeled cRNA was assessed using a NanoDrop ND-1000 spectrophotometer and an Agilent Bioanalyzer. Equal amounts of Cy3- or Cy5-labeled cRNA were hybridized to the Agilent Rat Oligo Microarray (Agilent Technologies) for 17 hours, prior to washing and scanning. Data were extracted from scanned images using Agilent's Feature Extraction Software (Agilent Technologies). Fluorophore reversal hybridizations were performed for 318 treated rats against time-matched control pools for both tissue types (blood and liver) for a total of 1,272 hybridizations. For microarray hybridizations performed on the Affymetrix platform, 1 μg of total RNA from the left liver lobe of each individual rat was labeled using the GeneChip One-Cycle Target Labeling kit (Affymetrix, Inc., Santa Clara, CA, USA) following the manufacturer's protocol. The amount and quality of the cRNA was assessed using a NanoDrop ND-1000 spectrophotometer and an Agilent Bioanalyzer. The cRNA was then fragmented and hybridized to the Rat Genome 230 2.0 Array (Affymetrix, Inc.) for 17 hours, prior to washing and scanning. Data were extracted from scanned images using GeneChip Operating Software (Affymetrix, Inc.). Hybridizations were performed on all treated and control animals for each compound in addition to pooled control samples for two of the compounds for a total of 432 hybridizations. These data are also available in CEBS under the CEBS ID# 001-00001-0020-000-4 [34].

### Data analysis

Gene expression data were loaded into the Rosetta Resolver^® ^Gene Expression Analysis System version 6.0.0.0.311. Fluorophore reversal hybridization data generated using the Agilent platform were combined using an error-weighted average for each individual animal. Data generated using the Affymetrix platform were loaded using the Rosetta error model for gene expression analysis [42]. Rosetta Resolver was used to perform SVM-based analysis using a Gaussian kernel function with three cross-validation loops, one-way error-weighted ANOVA (with Bonferroni multiple test correction) and PCA. SVMs were used to identify compound-specific classifiers on each tissue type (blood and liver) within a given dose/time group by randomly selecting two animals from each compound within a given dose/time group to which the SVM was performed to identify the classifiers. Therefore, each SVM was performed on a total of 16 animals (2 animals from each of the 8 compounds). A total of 18 SVMs were performed. The Agilent ProbeIDs for each of the classifiers that were identified in each of these SVMs are detailed in Additional data file 2. Hierarchical clustering was also performed in Rosetta Resolver using Ward's minimum variance hierarchical clustering with Euclidean distance as the similarity metric on ratios of gene expression data. GO enrichment analysis was performed using High-Throughput GoMiner [37,38].

## Abbreviations

ANOVA, analysis of variance; CEBS, Chemical Effects in Biological Systems; GO, Gene Ontology; PCA, principal component analysis; RBC, red blood cell; SVM, support vector machine; WBC, white blood cell.

## Authors' contributions

RSP, RWT, PH, EKL, KG, CJT, RDI, GAB, JTA, MLC and ANH conceived, designed, and supervised various aspects of the study. PEB, SOS and MLC participated in the in-life work/tissue collection and RNA purification. EKL and PH made substantial contributions to microarray processing. EKL, PH, PEB, SMW, RSP, PRB, KG, JMF, JTA, REW, BAM, DEM, and GAB contributed to the analysis and interpretation of the data. EKL drafted the manuscript. PH, RSP, PRB, KG, SOS, PH, JMF, BAM, DEM critically reviewed the manuscript. All authors read and approved the final manuscript.

## Additional data files

The following additional data are available with the online version of this paper. Additional data file 1 is a table detailing the histopathological scores as well as the clinical chemistry and hematology data that were generated for each animal in this project. Additional data file 2 is a table detailing the Agilent probe identifiers that resulted from the SVM analyses detailed in the Results section. Additional data file 3 is a table detailing the enrichment *p*-values that resulted from performing a GO enrichment analysis on the list of classifiers for each dose/time/tissue group. GO categories must have had at least five associated genes measured on the microarray in order to be included. Additional data file 4 is a table detailing all of the observations and measurements that were made during the in-life portion of the project. Additional data file 5 is a table detailing the scoring method that was used for the histopathological observations that were made in this project.

## Supplementary Material

Additional data file 1 Histopathological scores as well as the clinical chemistry and hematology data that were generated for each animal in this project. Click here for file

Additional data file 2 Agilent probe identifiers that resulted from the SVM analyses detailed in the Results section. Click here for file

Additional data file 3 Enrichment *p*-values that resulted from performing a GO enrichment analysis on the list of classifiers for each dose/time/tissue group. GO categories must have had at least five associated genes measured on the microarray in order to be included. Click here for file

Additional data file 4 Observations and measurements made during the in-life portion of the project. Click here for file

Additional data file 5 Scoring method used for the histopathological observations made in this project. Click here for file
