# Supplementary material for: Gene expression response in target organ and whole blood varies as a function of target organ injury phenotype
Source: Genome Biol. 2008 Jun 20;9(6):R100. doi: 10.1186/gb-2008-9-6-r100 (PMC2481421; doi:10.1186/gb-2008-9-6-r100)
Supplement: Additional data file 5 — Scoring method used for the histopathological observations made in this project. [file gb-2008-9-6-r100-S5.docx]

**Supplementary Table 2.** Scoring of Histopathology Diagnoses^a^

| **Diagnosis** | **Minimal (1)** | **Mild (2)** | **Moderate (3)** | **Marked (4)** |
| --- | --- | --- | --- | --- |
| Hepatocyte Necrosis^b^ | <5% | 5-25% | 26-50% | >50% |
| Hepatocyte Glycogen Depletion^b, c^ | <5% | 5-25% | 26-50% | >50% |
| Hepatocyte Degeneration^b^ | <5% | 5-25% | 26-50% | >50% |
| Mitosis  (number/10 high power fields) | 3 - 6 | 7 - 12 | 12 - 18 | >18 |
| Bile Duct Hyperplasia  (based on the number or percentage of portal tracts involved) | 4-10 | 10-20  (or 30% - 50%) | 50 – 80% | >80% |
| Cellular Infiltration^b, d^ | <5% | 5-25% | 26-50% | >50% |
| Hepatocyte Hypertrophy | Subjectively graded based on the extent of lobular involvement and the relative size of hepatocytes as compared with concurrent controls | | | |
| Hepatocyte Regeneration | Hepatocyte regeneration was diagnosed when there was a combination of elevated mitoses and other changes indicative of a regenerative response such as increased cell size, enlarged nuclei, cytoplasmic basophilia and prominent nucleoli. Grading was subjective and based on the number of regenerative hepatocytes relative to the total number of hepatocytes and the number of mitotic figures | | | |
| Hemorrhage^e^ | Diagnosed when there was a prominent and disproportionate amount of extravasated red blood cells compared to the degree of necrosis. Grading was subjective and based on the extent of lobular involvement. | | | |
| Congestion | Subjectively graded based on the width of sinusoidal dilation relative to controls | | | |
| Hepatocyte Apoptosis | Subjectively graded based on the number of apoptotic hepatocytes relative to concurrent controls | | | |
| Hepatocyte Fatty Change | Subjectively graded based on the relative amount of cytoplasmic lipid present in the entire section | | | |

^a^ Lesions were graded for relative severity using a four point grading scheme: grade 1 = minimal, grad 2 = mild, grade 3 = moderate, grade 4 = marked

^b^ These lesions were graded by estimating the percentage area affected in the entire section.

^c^ Diagnosed when hepatocytes appeared smaller with more homogenous and eosinophilic cytoplasm than concurrent controls. This finding frequently preceded hepatocellular degenerative lesions in this study set.

^d^ Cell type was classified based on the following criteria:

- mononuclear = > 70% mononuclear cells
- mixed = < 70% neutrophils and mononuclear cells
- neutrophilic = > 70% neutrophils

^e^ In some studies, the presence of hemorrhage was noted, but not scored. These animals are represented with a score of "5" in the Supplementary Table
